# Colo-Pro: a pilot randomised controlled trial to compare standard bolus-dosed cefuroxime prophylaxis to bolus-continuous infusion–dosed cefuroxime prophylaxis for the prevention of infections after colorectal surgery

**DOI:** 10.1007/s10096-018-3435-z

**Published:** 2018-12-05

**Authors:** Andrew Kirby, Eduardo Asín-Prieto, Flora Agnes Burns, Duncan Ewin, Kavi Fatania, Mithun Kailavasan, Saira Nisar, Agamemnon Pericleous, Iñaki F. Trocóniz, Dermot Burke

**Affiliations:** 1 Old Medical School, Leeds General Infirmary, Leeds Teaching Hospitals NHS Trust, Leeds, LS1 3EX UK; 2 University of Leeds, Leeds, LS3 1EX UK; 3 Pharmacometrics and Systems Pharmacology, School of Pharmacy and Nutrition, University of Navarra, Irunlarrea, 1, 31008 Pamplona, Spain; 4 IdiSNA, Navarra Institute for Health Research, Irunlarrea, 3, 31008 Pamplona, Spain

**Keywords:** Antibiotic, Bolus, Cefuroxime, Colorectal, Continuous, infusion, Prophylaxis

## Abstract

Standard bolus-dosed antibiotic prophylaxis may not inhibit growth of antibiotic resistant colonic bacteria, a cause of SSIs after colorectal surgery. An alternative strategy is continuous administration of antibiotic throughout surgery, maintaining concentrations of antibiotics that inhibit growth of resistant bacteria. This study is a pilot comparing bolus-continuous infusion with bolus-dosed cefuroxime prophylaxis in colorectal surgery. This is a pilot randomised controlled trial in which participants received cefuroxime bolus-infusion (intervention arm) targeting free serum cefuroxime concentrations of 64 mg/L, or 1.5 g cefuroxime as a bolus dose four-hourly (standard arm). Patients in both arms received metronidazole (500 mg intravenously). Eligible participants were adults undergoing colorectal surgery expected to last for over 2 h. Results were analysed on an intention-to-treat basis. The study was successfully piloted, with 46% (90/196) of eligible patients recruited and 89% (80/90) of participants completing all components of the protocol. A trialled bolus-continuous dosing regimen was successful in maintaining free serum cefuroxime concentrations of 64 mg/L. No serious adverse reactions were identified. Rates of SSIs (superficial and deep SSIs) were lower in the intervention arm than the standard treatment arm (24% (10/42) vs. 30% (13/43)), as were infection within 30 days of operation (41% (17/43) vs 51% (22/43)) and urinary tract infections (2% (1/42) vs. 9% (4/43)). These infection rates can be used to power future clinical trials. This study demonstrates the feasibility of cefuroxime bolus-continuous infusion of antibiotic prophylaxis trials, and provides safety data for infusions targeting free serum cefuroxime concentrations of 64 mg/L. Trial registration: NCT02445859.

**Electronic supplementary material:**

The online version of this article (10.1007/s10096-018-3435-z) contains supplementary material, which is available to authorized users.

## Introduction

Colorectal surgery is a common procedure, with approximately 100,000 operations annually within England, with 18–27% developing a surgical site infection (SSI) [1–6]. SSIs are a major healthcare concern as they are associated with increased morbidity, mortality, and cost [7]. In an attempt to prevent superficial and deep SSIs, antibiotic prophylaxis is given peri-operatively, normally as a bolus dose within the hour before surgery. Antibiotic prophylaxis is effective; when initially introduced for colorectal surgery, it reduced superficial and deep SSIs rates from 40 to 10% [8]. Recent data, however, indicate that SSI rates have increased [2–6, 8]. A potential reason for increased SSI rates may be suboptimal dosing of standard antibiotic prophylaxis, secondary to increasing rates of obesity and growing antimicrobial resistance [9, 10]. In patients undergoing colorectal surgery, one study identified that 20% of patients were colonised with antibiotic resistant Enterobacteriaceae, a genus associated with approximately 80% of SSIs after colorectal surgery [11]. Furthermore, standard bolus dosing has been reported as not achieving concentrations believed to be effective for preventing SSIs in operations lasting longer than 2 h [12]. A potential way of improving the effectiveness of prophylaxis is by targeting antibiotic concentrations throughout an operation at concentrations more likely to inhibit the growth of bacteria classified as resistant, by using a bolus dose of antibiotic, followed by a continuous infusion during surgery. We therefore undertook a pilot trial (Colo-Pro) of antibiotic prophylaxis administered as a bolus-continuous infusion, targeting a serum concentration able to inhibit both susceptible and resistant Enterobacteriaceae. The aims of this study were (1) to determine the feasibility of recruiting and following-up colorectal patients in a trial comparing bolus vs. bolus-continuous infusion of prophylactic cefuroxime, and (2) to determine if a bolus-continuous dosing regimen could achieve targeted serum levels of antibiotic through operations without serious adverse reactions, and (3) to describe outcome rates in the recruited study population.

## Materials and methods

### Design

A pilot, randomised controlled, parallel, single-blinded, single-centre, phase II/III trial was conducted in which patients were allocated in a 1:1 ratio to receive either cefuroxime bolus-infusion or standard bolus-dose antibiotic prophylaxis before colorectal surgery. This was an external pilot trial. All participants also received 500 mg of intravenous metronidazole. Ethical approval was obtained from the Health Research Authority (Reference 15/YH/0260), the protocol was reviewed by the Medical Health Research Authority (MHRA), and the study was conducted according to Good Clinical Practice standards with all patients providing informed consent. The trial was registered at Clinical trials.gov: NCT02445859.

### Participants

Patients were eligible if they were adults (≥ 18 years) undergoing colorectal surgery (incision, excision, or anastomosis of the large bowel, including anastomosis of small to large bowel) expected to last for more than 2 h. Patients were excluded if they were pregnant, had a cephalosporin allergy or penicillin hypersensitivity, coumarin treatment, concurrent use of probenecid, or a creatinine clearance below 40 mL/min. Participants were recruited from Leeds Teaching Hospitals NHS Trust from August 2015 to April 2017. Participants were identified and consented on their day of surgery on admitting surgical wards. Participants were enrolled and allocated to their intervention by research doctors (FAB, AP, MK, KF, SN).

### Randomisation

Randomisation was carried out by generating two lists of random numbers using an online sequence generator (https://www.random.org/sequences/). A person not involved in the trial produced sealed envelopes containing randomisation assignments. Patients were randomised prior to surgery. Patients were blind to the allocation; however, allocation information was known to the surgeon and anaesthetist. Research doctors completed follow-up after randomisation and were not blinded. Patients were randomised in theatres. An operating surgeon confirmed that surgery should be completed prior to the patient being randomised. Recruitment ended when 90 patients had been randomised.

### Standard dosing

Intravenous (IV) cefuroxime 1.5 g bolus administered four-hourly throughout surgery. The first dose was given within 1 h of surgery.

### Intervention dosing

Cefuroxime bolus-continuous dosing was based on targeting non-protein bound (free) serum concentrations of antibiotic at 64 mg/L throughout surgery. This was intended to ensure all patients achieved and maintained serum concentrations of at least 4 × 16 mg/L throughout surgery. This concentration, 16 mg/L, has been reported as the MIC90 (minimum inhibitory concentration 90 (the lowest concentration of antibiotic at which 90% of a population growth is inhibited)) for Enterobacteriaceae based on a clinical study of patients undergoing colorectal surgery at Leeds Teaching Hospitals [11]. Achieving 4 × the MIC has been associated with maximal antimicrobial efficacy [13]. Two regimens to achieve these concentrations were evaluated. One regimen was formula-based (non-compartment model) and the second was a population pharmacokinetic two-compartment model (compartment model). Both models used pharmacokinetic parameter estimates identified previously] [12, 14]. As a safety measure, to limit overall cefuroxime exposure, no more than 6 h of continuous infusion was administered. In cases where the duration of surgery was longer than 6 h, the infusion was stopped and the dosing regimen reverted to a four-hourly bolus dose from hour 10 of surgery. The loading dose was administered within the hour before surgery, with initiation of the continuous infusion before surgery. Further details of intervention dosing regimens are provided in Online Resource 1.

### Data collection and management

Creatinine clearance was estimated for each patient using the Cockcroft–Gault equation [15]. Data such as co-morbidities, dose and timing of cefuroxime administration, timing of blood sample collection, incision, and closure time were recorded prospectively.

### Sample size calculation

A formal power calculation was not required as this was a pilot study. However, to assess the feasibility of the trial design and to obtain 60 patients with analysable pharmacokinetic data, a recruitment target of 90 patients was set, assuming blood samples were collected from two thirds of patients [16].

### Outcomes

The main outcomes in this trial are related to trial feasibility. The main outcomes assessed are therefore recruitment rates, adherence to allocated interventions, and protocol completion rates. Protocol violations will therefore be reported and described.

For the purposes of facilitating estimations of clinical outcomes in a subsequent phase three trial, clinical outcomes were determined. Clinical outcomes measured were the rate of Surgical Site Infection (SSI), with superficial and deep SSIs included, within 30 days of operation. SSI definitions were the Centre for Disease Control’s (CDCs) National Nosocomial Infections Surveillance (NNIS) criteria for defining SSIs [17, 18]. SSIs were formally assessed on approximately day 5 and day 30 post-operatively by the research team. These assessments were carried out in person when patients were in-patients and by telephone using a structured questionnaire when an outpatient [18]. All in-patient infections, organ space SSIs, urinary tract infections, and *Clostridium difficile* infections within 30 days of operation (according to standard definitions [19]) were recorded. An outcome of infection within 30 days of surgery was assigned if a patient had any i.e. one or more, infections diagnosed in this time period. Additionally, days of hospitalisation within 30 days of operation and mortality after operation at 30 days and 1 year were recorded. Serious adverse reactions were defined as an event that was serious and believed with reasonable probability to be due to the trial treatment.

### Data analysis

Data were assessed by an intention-to-treat analysis; patients with all outcome data missing were excluded from analysis. In patients with incomplete outcome data, the last observation was carried forward [20]. Additional analyses included characterisation of antibiotic resistant Enterobacteriaceae colonising the colon pre-operatively, and a pharmacokinetic analysis of intra-operative serum cefuroxime concentrations. The CONSORT 2010 statement: extension to randomised pilot and feasibility trials was considered in the analysis of the data; see Online Resource 2 [21].

### Microbiology sample processing

Antibiotic effectiveness is related to antibiotic concentrations in patients relative to the susceptibility of bacteria. To determine the susceptibility of colonic Enterobacteriaceae, which we considered to be the most likely cause of post-operative SSIs, rectal or stoma swabs were collected from patients pre-operatively [18]. Swabs were screened for Enterobacteriaceae resistance by two methods: the first isolated the Enterobacteriaceae strain that was numerically predominant, and the second isolated the most antibiotic resistant strain. The predominant strain was identified by inoculating a CLED agar plate with the swab, streaking for isolated colonies, and determining the minimum inhibitory concentration (MIC) of these colonies from the terminal streak. To identify the most resistant organism, a CLED agar plate was swabbed for confluent growth and a 30-μg cefuroxime antibiotic disc (Oxoid) was placed in the centre. The growth closest to the disc was cultured to purity, and MICs were determined. MALDI-TOF and a 0.16 to 256 mg/L cefuroxime gradient strip (biomerieux) were used to determine species and MICs respectively. Resistance was defined as cefuroxime MIC > 8 mg/L [22].

### Antibiotic serum concentration processing

Blood samples were collected intra-operatively, up to four samples per patient throughout surgery, either via venepuncture or via an intravascular catheter. Blood samples were stored on ice intra-operatively, then, after centrifugation at 3000 rpm for 10 min at 5 °C. Serum was stored at − 70 °C until testing. Total serum cefuroxime concentrations were measured by high-performance liquid chromatography, which was performed on a Hypersil 5ODS column (HPLC Technology Ltd., Macclesfield, UK) using a mobile phase of methanol:water:phosphoric acid (25:74:1) and with detection by UV absorbance at 254 nm [23]. Samples were diluted 1:1 with acetonitrile, centrifuged at 5000*g* and a volume of 10 μL of the supernatant injected into the chromatograph. Quantification was by the external standard method with intra- and inter-assay precision (CV) below 5%.

## Results

### Recruitment

From August 2015 to April 2017 of 262 patients screened for study entry, 196 were eligible and 90 provided consent. This gave a recruitment rate of 46%. Of the 90 patients providing consent, 45 were randomised to the standard treatment group and 45 to the intervention group.

### Protocol adherence

There were 10 protocol violations: In the standard treatment arm, after randomisation, two patients did not undergo eligible colorectal surgery. In the intervention treatment arm, after randomisation, three patients did not undergo eligible colorectal surgery and two did not receive the allocated intervention. Three patients in the intervention arm received eligible colorectal surgery, the allocated intervention, and hospital in-patient follow-up, but did not complete all components of 30-day follow-up. See Online Resource 3 for a patient flow diagram. Of the 40 patients who received the intervention dosing, 18 received the non-compartment model dosing and 22 the compartment model dosing.

### Baseline characteristics of randomised groups

Both standard treatment and intervention groups had comparable baseline characteristics including: age (59 vs. 61 years), weight (78 vs 79 kg), rectal resection rate (54 vs 52%), ASA score of ≥ 3 (16 vs. 21%), NNIS (National Nosocomial Infections Surveillance risk index) scores (NNIS = 1, 60% vs. 57%), and operation types (open surgery, 30% vs 29%) (Table 1).

**Table 1:** Summary of patient characteristics for per protocol analysable patients

		Standard dosing (*n* = 43)	Intervention dosing (*n* = 42)
Sex	Male	56% (24/43)	64% (27/42)
Age	Mean, years	59	61
Weight	Mean, kg	78	78
Indication for surgery	Cancer	72% (31/43)	79% (33/42)
	Crohn’s	14% (6/43)	7% (3/42)
	Ulcerative colitis	9% (4/45)	14% (6/42)
	Other	9% (4/45)	7% (3/42)
Rectal resection	Yes	54% (23/43)	52% (22/42)
Chemotherapy within 12 months of surgery	Yes	23% (10/43)	21% (9/42)
Radiotherapy within 12 months of surgery	Yes	26% (11/43)	24% (10/42)
Bowel preparation^a^	Yes	42% (18/43)	31% (13/42)
ASA 3/4/5^b^	Yes	16% (7/43)	21% (9/42)
Operation over 3 h	Yes	58% (25/43)	52% (22/42)
Charlson score	Mean	2.1	2.7
Wound classification	Clean contaminated	100% (43/43)	98% (41/42)
NNIS^c^	0	33% (14/43)	33% (14/42)
	1	61% (26/43)	57% (24/42)
	2	7% (3/43)	5% (2/42)
	3	0	5% (2/42)
Stoma formed	Yes	47% (20/43)	31% (13/42)
Surgical type	Laparoscopic	56% (24/43)	64% (27/42)
	Open	30% (13/43)	29% (12/42)
	Robotic	7% (3/43)	2% (1/42)
	Lap to open	7% (3/43)	5% (2/42)
Drain inserted	Yes	44% (19/43)	43% (18/42)

^a^Pre-operative bowel preparation = oral formulations or rectal enema

^b^ASA = American Society of Anaesthesiologists physical status classification

^c^NNIS = National Nosocomial Infections Surveillance [NNIS] basic SSI risk index

### Clinical outcomes

Surgical site infection was found to be present in 30% (13/43) of standard dosing treatment patients and 24% (10/42) of patients receiving the intervention dosing treatment. Infection within 30 days of surgery was detected in 51% of patients in the standard dosing group, and in 41% of the intervention dosing group. There was only one death, with no deaths in either group within the 30 days after operation. Organ space infections occurred in 5% (2/43) of patients in the standard treatment dosing group and in 14% (6/42) of the intervention dosing group. Urinary tract infections occurred less commonly in the intervention group at 2% (1/42) compared to the standard treatment group at 9% (4/43). Outcomes are presented in Table 2.

**Table 2:** Outcome measures associated with intervention and standard dosing regimens per protocol analysis

	Standard dosing (*n* = 43)	Intervention dosing (*n* = 42)
SSI-S^a^ within 30 days of surgery	30% (13/43)	24% (10/42)
Infection within 30 days of surgery	51% (22/43)	41% (17/43)
Mean length of stay (days)	9.9	9.8
Death within 30 days of surgery	0% (0/43)	0% (0/42)
Death within 365 days of surgery	2% 1/43	0% 0/42)
SSI-O^b^ within 30 days of surgery	5% (2/43)	14% (6/42)
UTI^c^ within 30 days of surgery	9% (4/43)	2% (1/42)

^a^SSI-S: superficial surgical site infection

^b^SSI-O: organ space surgical site infection

^c^UTI: urinary tract infection

### Adverse reactions

No serious adverse reactions were identified; specifically, no cases of *Clostridium difficile* infection were identified within 30 days of surgery.

### Pharmacokinetics

**Fig. 1:**
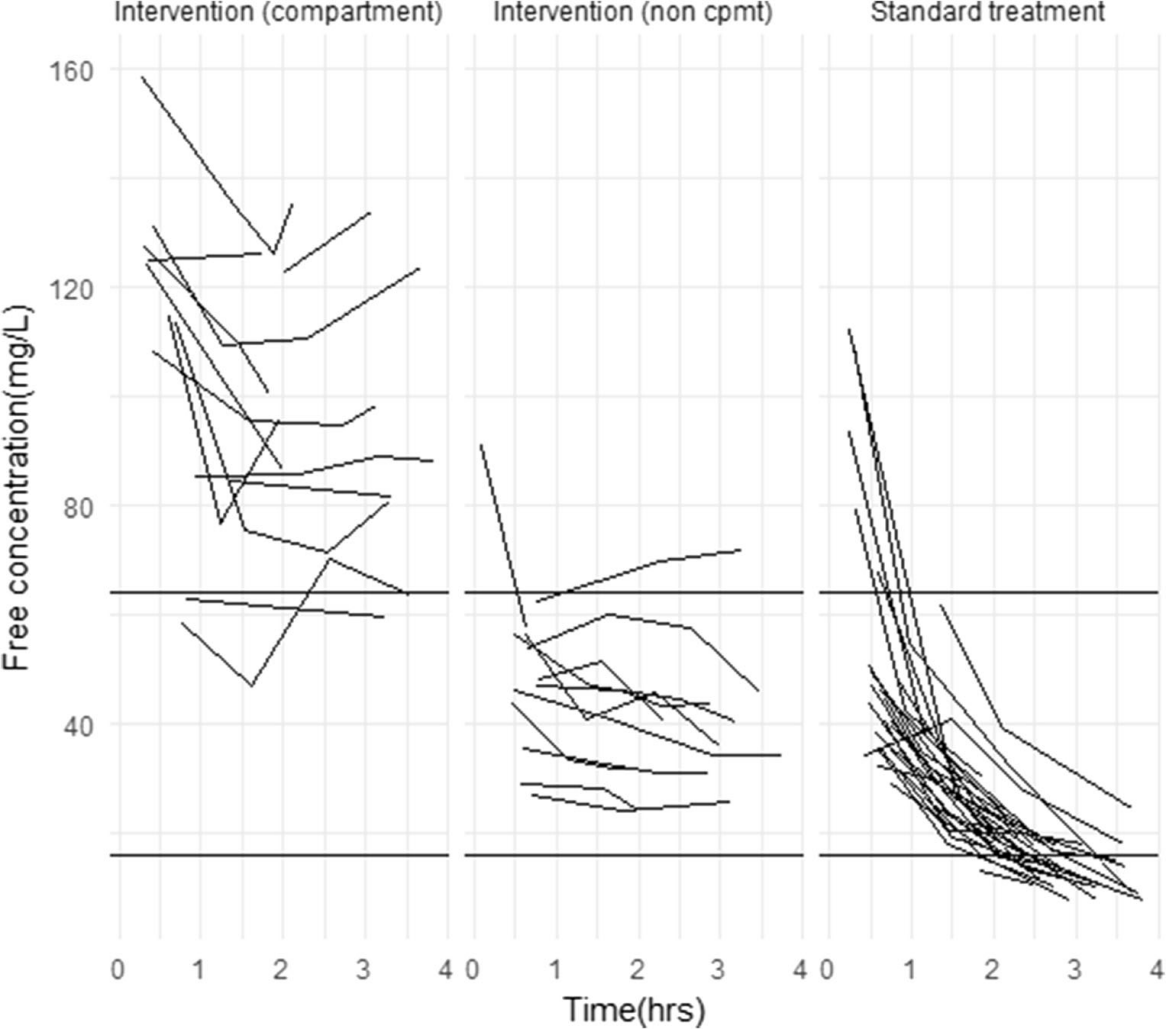
Free serum cefuroxime concentrations for 58 patients according to intervention treatment (compartment and non-compartment) and standard treatment dosing regimens. Horizontal lines represent 64 mg/L and 16 mg/L

A total of 58 patients had intra-operative blood samples collected. Based on a protein binding of 40%, free serum cefuroxime concentrations were determined [12]. These demonstrate that targeted concentrations of 64 mg/L throughout surgical procedures were achieved by the compartment-based model intervention regimen in 11/13 (85%) of patients. The non-compartment model maintained higher concentrations than standard bolus dosing (Fig. 1) but did not achieve targeted concentrations throughout surgery in any studied patients (0/10).

### Antibiotic susceptibility testing

The MIC was different depending upon the susceptibility test performed, with the screen for more antibiotic resistant bacteria identifying bacteria with a higher MIC50 (2 vs 1.5 mg/L) and higher MIC90 (12 vs 3 mg/L), see Table 3. There was no identified relationship between MIC and outcome.

**Table 3:** Microbiology and antibiotic susceptibility testing results from pre-operative colonic samples

	Bacterial species identified				Susceptibility to cefuroxime (mg/L)			
	*E. coli*	*Klebsiella* spp.	Other Enterobacteriaceae	None	MIC50	MIC90	% resistant^a^	Geometric mean
Predominant	67/90	6/90	8/90	9/90	1.5	3	3% (3/90)	2.1 (*n* = 81)
Resistant	52/90	9/90	19/90	11/90	2	12	11% (10/90)	3.5 (*n* = 80)

^a^Susceptibility defined as a cefuroxime breakpoint of > 8 mg/L

## Discussion

We have successfully demonstrated the feasibility of performing a trial of cefuroxime bolus-continuous infusion versus standard bolus cefuroxime antibiotic prophylaxis. This conclusion is based on a recruitment rate of 46%, which we consider good due to the complex and multidisciplinary setting in which recruitment was completed. In this trial, as the treatment was finished by the end of a patient’s surgical procedure, there were no issues related to adherence. Randomisation was successfully completed, and there were only a limited number of patients whose surgical procedures were not carried out. Future studies should aim to randomise at the last available opportunity, and confirm surgical procedures with the most senior member of the surgical team. The single blinding used in the trial was successfully piloted, but future studies should aim to also blind the patients’ clinical teams as well as outcome assessors. There were occasions when a safety concern in relation to low body weight and reduced renal function resulted in non-allocated antibiotic prophylaxis being administered. Guidance within the protocol related to these specific issues could prevent these protocol violations.

In assessing the intervention bolus-continuous dosing regimens, the compartment model–based dosing regimen, which was adjusted for renal function, was the more effective model and achieved target concentrations in > 80% of patients as predicted. This compartment based–dosing regimen is therefore suitable to be used in a clinical trial targeting free serum concentrations of 64 m/L of cefuroxime. The demonstrated feasibility of this trial is important, as it has been shown that antibiotic concentrations at both the start and the end of surgery are important predictors of clinical effectiveness [24]. The bolus-continuous infusion approach to prophylaxis is a strategy that can achieve desired concentrations of antibiotic throughout surgery, including the end of surgery, across multiple surgical procedures, and for multiple antibiotics, making the bolus-continuous infusion approach highly generalisable.

Rates of SSIs (superficial and deep SSIs) were lower in the intervention group, as were any infections and rates of urinary tract infection. These lower rates are consistent with the aim of the intervention, which is to reduce post-operative infections. As the difference in any infection between intervention groups was higher than the difference between SSIs, infection within 30 days of operation may be considered as the optimal outcome measure in future trials. This approach is supported by previous data that show that antibiotics with a long half-life e.g. ceftriaxone, which act like a continuous infusion of a short half-life antibiotic e.g. cefuroxime, reduce post-operative respiratory tract, surgical site, and urinary tract infections [25]. However, these long half-life antibiotics are currently avoided in clinical practice over concerns relating to increased risks of *Clostridium difficile* infection. All three patients lost-to follow-up at day 30 had an infection identified before their day 30 review. A future study could therefore consider using an intention-to-treat analysis with “the last observation carried forward” method to deal with this as done in this study analysis, or could consider including an outcome measure assessment after a shorter time period e.g. 14 days.

The rate of organ space surgical site infection was higher in the intervention group. Considering known rates of organ space infection it is likely this reflects a lower than expected rate of these events in the standard treatment group [2]. Alternatively, it has been suggested that antibiotics have the potential to select for bacteria with collagenase activity, with the ability to cause anastomotic leak [26]. Higher doses of antibiotic associated with the intervention treatment could therefore be consistent with increased selective pressure on the bacteria, and so increased anastomotic leak and subsequent organ space infection. No mortality was seen in the 30 days after the intervention in either treatment group.

The compartment model intervention was successful in obtaining serum concentrations targeted at 64 mg/L for the duration of surgery. This study was performed on a population of patients with high rates of cefuroxime susceptible Enterobacteriaceae colonisation of the colon, with resistance detected in 3–11% of patients, depending on the method of resistance detection used. It is possible that the effectiveness of the intervention may be dependent upon the rate/characteristics of Enterobacteriaceae resistance.

In summary, completion of this feasibility study suggests that a large pragmatic trial into bolus-continuous infusion of cefuroxime prophylaxis can be completed. As rates of antibiotic resistance increase the need for such trials is becoming more urgent.

## Electronic supplementary material

ESM 1 (DOCX 34 kb)

ESM 2 (DOCX 50 kb)

ESM 3 (DOCX 50 kb)

## Data Availability

The datasets generated during and analysed during the current study are available from the corresponding author on reasonable request.
