# Supplementary material for: Colo-Pro: a pilot randomised controlled trial to compare standard bolus-dosed cefuroxime prophylaxis to bolus-continuous infusion–dosed cefuroxime prophylaxis for the prevention of infections after colorectal surgery
Source: Eur J Clin Microbiol Infect Dis. 2018 Dec 5;38(2):357–63. doi: 10.1007/s10096-018-3435-z (PMC6514115; doi:10.1007/s10096-018-3435-z)
Supplement: Supplementary file 1 — (DOCX 34 kb) [file 10096_2018_3435_MOESM1_ESM.docx]

**Intervention dosing regimens estimation**

In order to achieve a pharmacodynamic target of free serum cefuroxime concentration 4 x MIC90 (fC_Target_ = 64mg/L) during the entire surgical procedure, intervention arm patients received a loading dose followed by a continuous infusion during the surgery (up to 6 hours).

The doses were estimated using two different approaches:

- Non-compartment based model
- Compartmental analysis based model

**Non-compartment based model:** This dosing regimen for both the bolus loading dose and continuous infusion dose was calculated using the following formulas and data [25]

- Bolus (loading dose) (mg) = target concentration of free drug (64mg/L) / 0.67^*^ x volume of distribution (L/kg) x body weight (kg) (volume of distribution = 0.19 L/kg, ^*^protein binding = 33%)
- Continuous (maintenance) infusion dose (mg/h) = target concentration of free drug (64mg/L) / 0.67 x total body clearance (L/h).
- The continuous infusion rate in renal impairment (creatinine clearance < 90 mL/min) (mg/h) = continuous infusion rate x (Creatinine clearance/100).

The resulting dosing regimens are shown in Table 4.

**Compartmental analysis based model**: The following expression was used to calculate the loading dose using pharmacokinetic parameters obtained from a population pharmacokinetic analysis in which the plasma concentration vs. time profiles of cefuroxime were described with a two compartments model [26], and assuming that the ratio between the areas under the unbound concentration vs. time curves in muscle and total plasma equals 1 [27].

$$Loading dose \left( mg \right)= \frac{{fC}_{Target}}{(1-f_{u})}\times V_{1}+{fC}_{Target}\times V_{2}$$

Where f_u_ is the typical unbound fraction in plasma of cefuroxime (f_u_ = 0.4 [28]) and V_1_ and V_2_ represent the apparent volume of distribution of the central and peripheral compartments respectively. Additionally, the maintenance infusion rate was estimated using the following equation:

$$Maintenance infusion rate \left( \frac{mg}{h} \right)=\frac{{fC}_{Target}}{\left( 1-f_{u} \right)} \times CL$$

Individual estimates of V_1_, V_2_, and CL, total plasma clearance, were generated using the values shown in table 5 applying a conservative approach in order to guarantee that the majority of the patient population (>80%) achieved cefuroxime concentrations higher than the target concentration based on the parameter distributions using the following equation.

$$P_{i}=P_{pop}\times\left( 1+\sqrt{\omega_{P}^{2}} \right)$$

Where P_i_ is the individual pharmacokinetic parameter value used in the above mentioned dose estimations, P_pop_, is the typical (same for all generated patients) population estimate, and $\omega_{P}^{2}$, is the estimated variance for the corresponding parameter (*P*) that reflects the discrepancy between P_i_ and P_pop_. Table 5 lists the typical population values of the pharmacokinetic parameters and their corresponding variances and co-variances. Please note that for the case of CL, there are two typical population values depending on the creatinine clearance.

As the reference pharmacokinetic model did not reveal body weight (between 40-110kg) as a covariate of cefuroxime pharmacokinetics, the intervention is therefore dosed by renal function (using the creatinine clearance as a discrete variable the middle value for each range of renal function, Table 4), for a maximum duration of 6 hours. In cases where the duration of surgery is longer than 6 hours, the infusion is stopped and the dosing regimen is reverted to a 4-hourly bolus dose from hour 10. The loading dose is administered within the hour before surgery, with initiation of the continuous infusion before surgery. The dosing regimen is shown in Table 4.

The therapeutic success of the calculated doses (expressed as probabilities of achieving the target concentration > 80%) were confirmed by visual inspection of simulated free cefuroxime concentration profiles of one thousand virtual patients depending on the renal function using ‘mlxR’ package on R version 3.2.0 [29].

Table 4: Cefuroxime dosing regimens for the intervention treatment

| Weight (kg) | Loading dose (mg) | Continuous infusion dose per hour: Based on creatinine clearance (ml/min) | | | | | |
| --- | --- | --- | --- | --- | --- | --- | --- |
|  |  | 40-50 | 50-60 | 60-70 | 70-80 | 80-90 | >90 |
| Non compartment model | | | | | | | |
| 30-40 | 617 | 128 | 157 | 185 | 214 | 242 | 285 |
| 40-50 | 793 | 165 | 202 | 238 | 275 | 312 | 367 |
| 50-60 | 970 | 202 | 246 | 291 | 336 | 381 | 448 |
| 60-70 | 1146 | 238 | 291 | 344 | 397 | 450 | 529 |
| 70-80 | 1322 | 275 | 336 | 397 | 458 | 519 | 611 |
| 80-90 | 1499 | 312 | 381 | 450 | 519 | 589 | 692 |
| 90-100 | 1675 | 348 | 426 | 503 | 580 | 658 | 774 |
| 100-110 | 1851 | 385 | 470 | 556 | 641 | 727 | 855 |
| Compartment model | | | | | | | |
| NA | 2331 | 723 | 867 | 1011 | 1155 | 1227 | 1227 |

Table 5: Parameter estimations based on cefuroxime model [26]

| **PARAMETER ESTIMATES** | |
| --- | --- |
| **CL** (L/h) = 8.77+ (CL_CR_ - 4.8) × 1.71  if (CL_CR_ ≥ 4.8 L/h) → **CL** (L/h) = 8.77  **V1** (L) = 11.27  **Q** (L/h) = 7.11  **V2** (L) = 7.30 | ω^2^_V1_ = 0.304  ω^2^_CL_ = 0.0966  Covariance (ω^2^_V1_, ω^2^_CL_) = 0.135 |
| CL: Total body clearance; CL_CR_: creatinine clearance; V1: apparent volume of distribution of the central compartment (plasma); Q: inter-compartment clearance; V2: apparent volume of distribution of the peripheral compartment; IIV: inter-individual variability. | |

**References**

1. Nascimento JW, Carmona MJ, Strabelli TM, Auler JO Jr, Santos SR (2007) Perioperative cefuroxime pharmacokinetics in cardiac surgery. Clinics (Sao Paulo) 62:257-60.
2. Asín-Prieto E, Soraluce A, Trocóniz IF, Campo Cimarras E, Sáenz de Ugarte Sobrón J, Rodríguez-Gascón A, Isla A (2015) Population pharmacokinetic models for cefuroxime and metronidazole used in combination as prophylactic agents in colorectal surgery: Model-based evaluation of standard dosing regimens. Int J Antimicrob Agents;45:504–511.
3. Barbour A, Schmidt S, Rout WR, Ben-David K, Burkhardt O, Derendorf H (2009) Soft tissue penetration of cefuroxime determined by clinical microdialysis in morbidly obese patients undergoing abdominal surgery. Int J Antimicrob Agents;34:231-235
4. European Committee on Antimicrobial Susceptibility Testing. Cefuroxime: Rationale for the clinical breakpoints, version 1.0, 2010. <http://www.eucast.org>
5. Marc Lavielle (2016) mlxR: Simulation of Longitudinal Data. R package version 3.0.0. Available at: http://CRAN.R-project.org/package=mlxR. Accessed 11 June 2018.

**Title**: Colo-Pro Pilot: A pilot randomised controlled trial to compare standard bolus dosed cefuroxime prophylaxis to bolus-continuous infusion dosed cefuroxime prophylaxis for the prevention of infections after colorectal surgery.

**Journal name**: European Journal of Clinical Microbiology and Infectious Diseases

**Authors**: Andrew Kirby^1,2^*, Eduardo Asín Prieto^3^, Duncan Ewin^2^, Flora Agnes Burns^1^, Agamemnon Pericleous^1^, Mithun Kailavasan^1^, Kavi Fatania^1^, Saira Nasir^1^, Iñaki F.Trocóniz^3^, Dermot Burke^1,2^.

**Affiliations**:

1-Leeds Teaching Hospitals NHS Trust, Leeds, UK

2-University of Leeds, Leeds LS3 1EX

3-Department of Pharmacy and Pharmaceutical Technology, University of Navarra, Pamplona, Spain

***Correspondence:** Dr Andrew Kirby, Old Medical School, Leeds General Infirmary, Leeds, LS1 3EX. Tel: 0113 3923929. E-mail: a.kirby@leeds.ac.uk
