# Supplementary material for: Colo-Pro: a pilot randomised controlled trial to compare standard bolus-dosed cefuroxime prophylaxis to bolus-continuous infusion–dosed cefuroxime prophylaxis for the prevention of infections after colorectal surgery
Source: Eur J Clin Microbiol Infect Dis. 2018 Dec 5;38(2):357–63. doi: 10.1007/s10096-018-3435-z (PMC6514115; doi:10.1007/s10096-018-3435-z)
Supplement: Supplementary file 2 — (DOCX 50 kb) [file 10096_2018_3435_MOESM2_ESM.docx]

**Colo-Pro Patient Flow Diagram**

## Enrollment

## Analysis

Analysed (n=43)

♦ Excluded from the analysis (did not receive eligible surgery and no clinical outcome data collected) (n=2)

Analysed (n=42)

♦ Excluded from the analysis (did not receive eligible surgery and unable to collect clinical outcome data) (n=3)

Lost to follow-up (give reasons) (n=0)

Discontinued intervention (n=0)

Lost to follow-up (unable to obtain complete 30 day follow up) (n=3)

Discontinued intervention (n=0)

## Follow-Up

Allocated to bolus regimen (n=45)

♦ Received allocated intervention (n=43)

♦ Did not receive allocated intervention (did not receive eligible surgery (n=2))

Allocated to bolus-continuous infusion (n=45)

♦ Received allocated intervention (n=40)

♦ Did not receive allocated intervention (did not receive eligible surgery (n=3), did not receive allocated treatment as reduced renal function (n=1), and low body weight (n=1)

Excluded (n=172)

♦  Not meeting inclusion criteria (n=66)

♦  Declined to participate (n=106)

## Allocation

Randomized (n=90)

Assessed for eligibility (n=262)

**Title**: Colo-Pro Pilot: A pilot randomised controlled trial to compare standard bolus dosed cefuroxime prophylaxis to bolus-continuous infusion dosed cefuroxime prophylaxis for the prevention of infections after colorectal surgery.

**Journal name**: European Journal of Clinical Microbiology and Infectious Diseases

**Authors**: Andrew Kirby^1,2^*, Eduardo Asín Prieto^3^, Duncan Ewin^2^, Flora Agnes Burns^1^, Agamemnon Pericleous^1^, Mithun Kailavasan^1^, Kavi Fatania^1^, Saira Nasir^1^, Iñaki F.Trocóniz^3^, Dermot Burke^1,2^.

**Affiliations**:

1-Leeds Teaching Hospitals NHS Trust, Leeds, UK

2-University of Leeds, Leeds LS3 1EX

3-Department of Pharmacy and Pharmaceutical Technology, University of Navarra, Pamplona, Spain

***Correspondence:** Dr Andrew Kirby, Old Medical School, Leeds General Infirmary, Leeds, LS1 3EX. Tel: 0113 3923929. E-mail: a.kirby@leeds.ac.uk
